# Modulation of Auxin Levels in Pollen Grains Affects Stamen Development and Anther Dehiscence in *Arabidopsis*

**DOI:** 10.3390/ijms19092480

**Published:** 2018-08-22

**Authors:** Hernán Salinas-Grenet, Ariel Herrera-Vásquez, Samuel Parra, Allan Cortez, Lilian Gutiérrez, Stephan Pollmann, Gabriel León, Francisca Blanco-Herrera

**Affiliations:** 1 Centro de Biotecnología Vegetal, Facultad de Ciencias de la Vida, Universidad Andres Bello, Santiago, RM 837-0186, Chile; h.salinasgrenet@gmail.com (H.S.-G.); asherrer@uc.cl (A.H.-V.); samuel.parra.a@gmail.com (S.P.); cortezvidal.a@gmail.com (A.C.); lilian.gm885@gmail.com (L.G.); gabriel@divulgacion.cl (G.L.); 2 Millennium Institute for Integrative Systems and Synthetic Biology (MIISSB), Santiago, Chile; 3 Centro de Biotecnología y Genómica de Plantas, Universidad Politécnica de Madrid (UPM)–Instituto Nacional de Investigación y Tecnología Agraria y Alimentaria (INIA), 28223 Pozuelo de Alarcón, Spain; stephan.pollmann@upm.es

**Keywords:** auxin, pollen, anther, *iaaL*, dehiscence

## Abstract

Auxin regulates diverse aspects of flower development in plants, such as differentiation of the apical meristem, elongation of the stamen, and maturation of anthers and pollen. It is known that auxin accumulates in pollen, but little information regarding the biological relevance of auxin in this tissue at different times of development is available. In this work, we manipulated the amount of free auxin specifically in developing pollen, using transgenic *Arabidopsis* lines that express the bacterial indole-3-acetic acid-lysine synthetase (*iaaL*) gene driven by a collection of pollen-specific promoters. The *iaaL* gene codes for an indole-3-acetic acid-lysine synthetase that catalyzes the conversion of free auxin into inactive indole-3-acetyl-l-lysine. The transgenic lines showed several abnormalities, including the absence of short stamina, a diminished seed set, aberrant pollen tubes, and perturbations in the synchronization of anther dehiscence and stamina development. This article describes the importance of auxin accumulation in pollen and its role in stamina and anther development.

## 1. Introduction

Auxins are phytohormones of major importance for plant growth and development. There is extensive evidence of how this plant hormone regulates different physiological processes, such as cell development, including cell division, differentiation, and elongation, wall plasticity, tropisms, apical dominance, senescence, and flower development [1,2]. Indole-3-acetic acid (IAA) is the most common naturally occurring active auxin in plants. However, the active free IAA comprises only up to 25% of the total amount of IAA in a cell, depending on the tissue and plant species investigated. The majority of cellular auxin is found in inactive forms, conjugated to either amino acids, sugars, or small peptides [3]. The metabolic control of the cellular auxin homeostasis through a tight regulation of de novo auxin biosynthesis, auxin degradation, and conjugation/deconjugation decisively orchestrates plant developmental processes [4].

The role of auxin over the course of flower development has great importance in the formation of reproductive organs [5]. For example, plants lacking the genes transport inhibitor response-1/auxin-binding F-box protein (*TIR/AFB*), responsible for the auxin perception, present flowers with precocious pollen maturation [6]. Additionally, mutant plants unable to express the auxin response factor 17 (*ARF17*) gene display aberrant phenotypes at the pollen level, including changes in the cell-wall pattern affecting its viability [7]. Also, two atypical members of the PIN auxin efflux carriers, *PIN5* and *PIN8*, suggested as regulators of auxin homeostasis in pollen, participate in the pollen development and morphology [8,9]. At the same time, it was demonstrated that the complex interplay between auxin biosynthesis is of utmost importance for the development of floral organs, especially for anther dehiscence and pollen viability [6]. Double mutants in the auxin biosynthesis-related *YUCCA* (flavin monooxygenase-like enzymes) family genes *YUC2* and *YUC6*, which are expressed during the early stages of anther and pollen development, present aberrant flower phenotypes, affecting stamina and anthers mainly. Accordingly, the *yuc2/yuc6* mutant plants are sterile [10]. On the other hand, plants expressing the bacterial indole-3-acetic acid-lysine synthetase (*iaaL*) gene driven by the *Arabidopsis thaliana* phosphatidylinositol monophosphate 5-kinase 1 (*PIP5K1*) promoter, which facilitates a tissue-specific expression in stamina filaments, showed shorter stamina filaments than those of wild-type plants, which is due to a reduced elongation of epidermal cells [11]. The iaaL enzyme catalyzes the inactivation of free IAA through its conjugation to the amino acid l-lysine. In addition, these plants also display a high rate of infertility. Pollen of these transgenic plants were unviable, because these pollen were unable to undergo the first round of pollen mitosis, generating aberrant pollen grain [11]. Further indirect evidence for an accumulation of IAA in flowers was provided by employing the auxin signaling reporter construct DR5:β-glucuronidase (GUS) [12]. The detailed histological analysis of the anthers at certain developmental stages determined the accumulation and dynamics of auxin in the tapetum, the middle layer, and the endothecium, which are the central tissues responsible for the dehiscence of the anther. An accumulation of auxin in the pollen during development was also observed [6]. Currently, the importance of auxin in floral development is demonstrated, but it is not known if the accumulation of auxin in the pollen affects the development of the flower. In this work, we demonstrate that the accumulation of auxin in the pollen grain plays an essential role for the stamina and anther development. Transgenic plants expressing the *iaaL* gene driven by a pollen-specific promoter in the DR5:GUS genetic background were produced and analyzed. The corresponding plants were characterized as having significantly decreased free IAA levels in the pollen, presumably through the enzymatic conversion of IAA to indole-3-acetyl-l-lysine (IAA-Lys). Consequently, the plants showed the loss of short stamina, as well as a loss of synchronization of stamina and anther development. Furthermore, they displayed defects in pollen tube development and had a smaller number of seeds. In summary, this work provides the first evidence for a role of auxin accumulation in the pollen grain, and how the deficiency of this hormone affects the proper development of the stamina and anthers.

## 2. Results

### 2.1. Auxin Signaling Is Activated in Developing Pollen Grains

**Figure 1:**
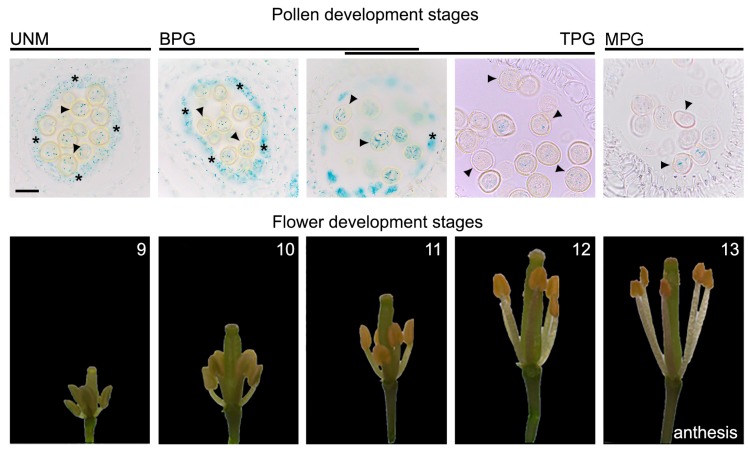
Auxin signaling is activated in pollen grain. Pollen from plants carrying the auxin transcriptional reporter DR5:β-glucuronidase (GUS) were evaluated. Histological GUS staining was observed during different stages of pollen development. UNM: uninucled microspore; BPG: bicellular pollen grain; TPG: tricellular pollen grain; MPG: mature pollen grain (upper panels, [14]). The corresponding floral development stages following the staging described by Bowman et al. (1994) [17] are shown (numbers, lower panels). Tapetum cells and pollen are indicated by asterisks and arrowheads, respectively. Scalebar: 20 μm.

Auxin is involved in virtually all plant developmental processes, including flower development. This study aimed to gain a deeper understanding of the role of auxin accumulation in pollen and its participation in the development of stamina and anthers. It is known that the developmental process of the anther and the pollen is synchronized; pollen is developed inside the anther in structures called locules, and four sporophytic cell layers encircle it: the tapetum, the middle layer, the endothecium, and the epidermis. Inside the locules, a diploid male meiocyte divides into a tetrad of four haploid microspores [13,14]. Each microspore, called a uninucleated microspore (UNM), undergoes asymmetric mitosis, denominated pollen mitosis I, resulting in two structurally and functionally different daughter cells: the small generative cell and the sizeable vegetative cell, called bicellular pollen (BCP). After this process, the generative nucleus is divided into another asymmetric mitosis (pollen mitosis II) to produce two sperm cells, whereas the vegetative cell no longer divides. At this moment, this structure is called tricellular pollen (TCP). After all these processes, the pollen is dehydrated and is called a mature pollen grain (MPG) which contains two haploid sperm cells and one haploid vegetative cell [13,14]. To characterize the auxin response dynamics during pollen development, histological sections of flowers from DR5:GUS reporter plants were analyzed. GUS assays were performed, and flower buds were separated from inflorescences and prepared for histological sections (Figure 1 and Figure S1). Deduced from the strong auxin signaling activity, the results suggest an accumulation of auxin in anther tissues that surround the pollen. Auxin signaling activity was detected in the tapetum and pollen during early floral developmental stages (8–9), where the pollen is present as a uninucleate microspore. The strong GUS staining in pollen is also visible during floral stages 9 to 11, where the pollen is present as bicellular and tricellular cells. Finally, during late floral stages (12–14), the mature pollen still presents GUS staining, suggesting that the auxin is present throughout pollen development, from uninuclear microspore to mature pollen grains. The stages of pollen development were determined from the tissues surrounding the pollen in the locules of the anther [14,15,16,17] (Figure 1). These results suggest an accumulation of auxin across all stages of pollen development, from early to late stages, independently of the anther tissues.

### 2.2. Genetic Impairment of Auxin Accumulation in Pollen Grain Leads to Stamen, Pollen, and Seed Phenotypes

After gaining evidence for an accumulation of auxin during the development of the pollen, the question arose as to whether this accumulation plays any role during flower development. Using the *iaaL* gene, which encodes for an IAA-amide synthetase that conjugates IAA with l-lysine, thereby inactivating the hormone, we aimed to manipulate free IAA content exclusively in pollen. To control the expression of *iaaL*, promoters of pollen-specific genes were used [18,19,20,21]. The promoters of the selected genes, sugar transporter 2 (*STP2*), sugar transporter 9 (*STP9*), pollen-specific gene 2 (*PSG2*), and phosphatase and tensin homolog deleted on chromosome 10 (*PTEN1*), drive differential gene expression over the course of pollen grain development (Figure S2). Based on their characteristics, the promoters were classified as an early pollen promoter (_p_EPP/*STP2*), intermediate pollen promoter (_p_IPP/*STP9*), intermediate pollen promoter II (_p_IPP2/*PSG2*), and late pollen promoter (_p_LPP/*PTEN1*).

**Figure 2:**
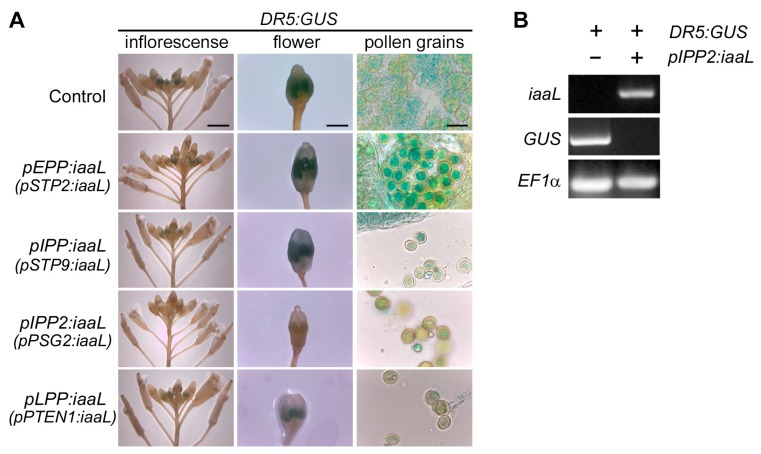
Flowers of transgenic plants expressing the indole-3-acetic acid-lysine synthetase (*iaaL*) gene controlled by floral tissue-specific promoters show a decrease in the expression of the auxin sensitive reporter DR5:GUS during different pollen developmental stages. (**A**) GUS activity was evaluated on inflorescences (left panels, scalebar: 20 mm), flowers (central panels, scalebar: 1 mm), and pollen grains (right panels, scalebar: 20 μm). The control plants DR5:GUS (upper panels) and the double transgenic plants carrying the DR5:GUS construct and the different promoters controlling the expression of the *iaaL* gene are shown: early pollen promoter (_p_EPP:*iaaL*); two intermediate pollen promoters (_p_IPP:*iaaL*; _p_IPP2:*iaaL*), and late pollen promoter (_p_LPP:*iaaL*). (**B**) The expression of transgenic constructs was evaluated with RT-PCR using RNA from pollen of DR5:GUS and DR5:GUS/_p_IPP2:*iaaL* plants. The expressions of *iaaL* and *GUS* genes were evaluated. The *EF1a* gene was used as housekeeping gene. Gene promoter names are indicated in parentheses.

The promoters were cloned, fused to the *iaaL* gene, and then introduced into the DR5:GUS genetic background. Firstly, the expression of the *iaaL* gene in the transgenic lines was confirmed by analyzing *iaaL* gene expression in the transgenic lines using semiquantitative RT-PCR (Figure S3). Next, the effect of the presumed reduction in free auxin levels in the pollen was assessed. To achieve this, we performed a GUS activity assay in all these transgenic plants. As shown in Figure 2, the transgenic lines expressing _p_EPP:*iaaL*, _p_IPP:*iaaL*, and _p_LPP:*iaaL* in the DR5:GUS background displayed a minor difference compared to the DR5:GUS parental line. Nevertheless, for plants expressing _p_IPP2:*iaaL* (Figure 2A), a clear reduction in the activity of the *GUS* reporter gene, specifically in the pollen, was detected. To confirm this observation, RT-PCR experiments using complementary DNA (cDNA) from pollen were performed. Expression of the *iaaL* gene was observed in pollen of the _p_IPP2:*iaaL* line, while no *GUS* expression was detected (Figure 2B). This suggests that the amount of free IAA in pollen is substantially reduced in pollen of the _p_IPP2:*iaaL* line. Thus, all subsequent experiments focused on the transgenic _p_IPP2:*iaaL* line.

**Figure 3:**
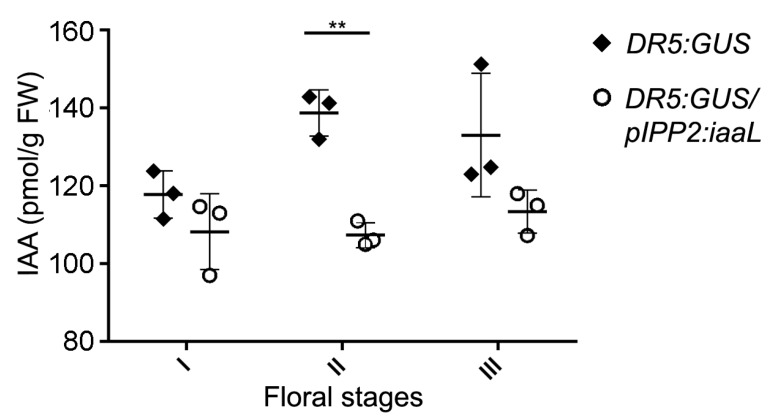
Transgenic _p_IPP2:*iaaL* plants show a decrease in auxin content during the intermediate floral developmental stage. Different flowers stages were collected from plants DR5:GUS (black diamonds) and DR5:GUS/_p_IPP2:*iaaL* (white circles) to evaluate the content of auxin (pmol IAA/g FW) using gas chromatography–tandem mass spectrometry analysis (GC–MS/MS). Floral stages are indicated (stage I: 7–8, stage II: 9–10, and stage III: 11–13). Asterisks indicate significant differences comparing the auxin levels of DR5:GUS/_p_IPP2:*iaaL* and the control DR5:GUS transgenic line according to a two-way ANOVA and Bonferroni post test (** *p* < 0.01, *n* = 3).

The next goal was to measure the amount of free IAA in this line in comparison to DR5:GUS control plants (Figure 3). Flowers of transgenic plants _p_IPP2:*iaaL* and DR5:GUS were divided into three developmental stages (stages 7–8 (I), stages 9–10 (II), and stages 11–13 (III); Figure S4) [17] and analyzed separately for auxin levels.

The mass spectrometric analysis of the lines did not show significantly different auxin levels at the early and late stages *(*Figure 3, I and II). Interesting, however, is the observation that the spatiotemporal expression of *PSG2* (_p_IPP2) occurs during the intermediate stages (9–10), which is suggested by the significant reduction (23.63%) in the amount of auxin during the intermediate stages of pollen development, relative to the DR5:GUS control (Figure 3, II).

Taken together, the results demonstrate that the _p_IPP2:*iaaL* transgenic line is characterized by low free-auxin contents during intermediate stages of flower development (floral stages 9–10 (II)), as evidenced directly by the mass spectrometric analysis of IAA and indirectly by the analysis of the *GUS* reporter gene driven by the synthetic auxin responsive DR5 promoter [12].

**Figure 4:**
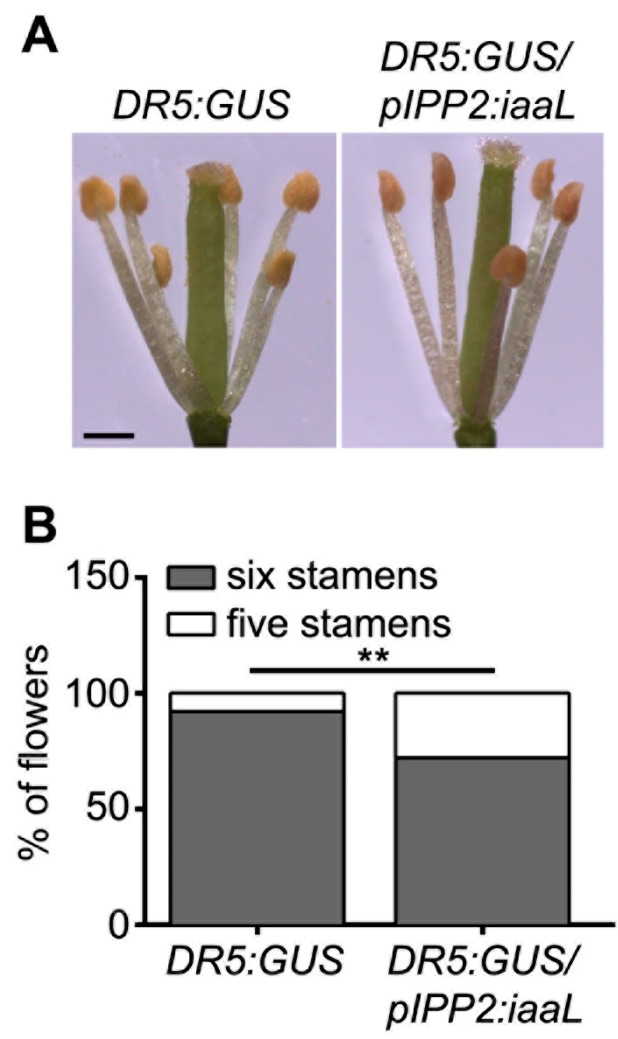
Flowers from transgenic DR5:GUS/IPP2:*iaaL* plants with diminished auxin content show loss of short stamina. (**A**) Representative flowers of DR5:GUS and DR5:GUS/_p_IPP2:*iaaL* plants (scalebar: 1 mm). (**B**) Quantification of the number of stamina per flower was performed in 200 flowers per genotype. The results were grouped as flowers with five (white boxes) or six stamina (gray boxes). Asterisks indicate significant differences comparing number of stamina of both DR5:GUS and DR5:GUS/_p_IPP2:*iaaL* genotypes according to a Fisher’s exact test (*p* < 0,01, *n* = 200).

**Figure 5:**
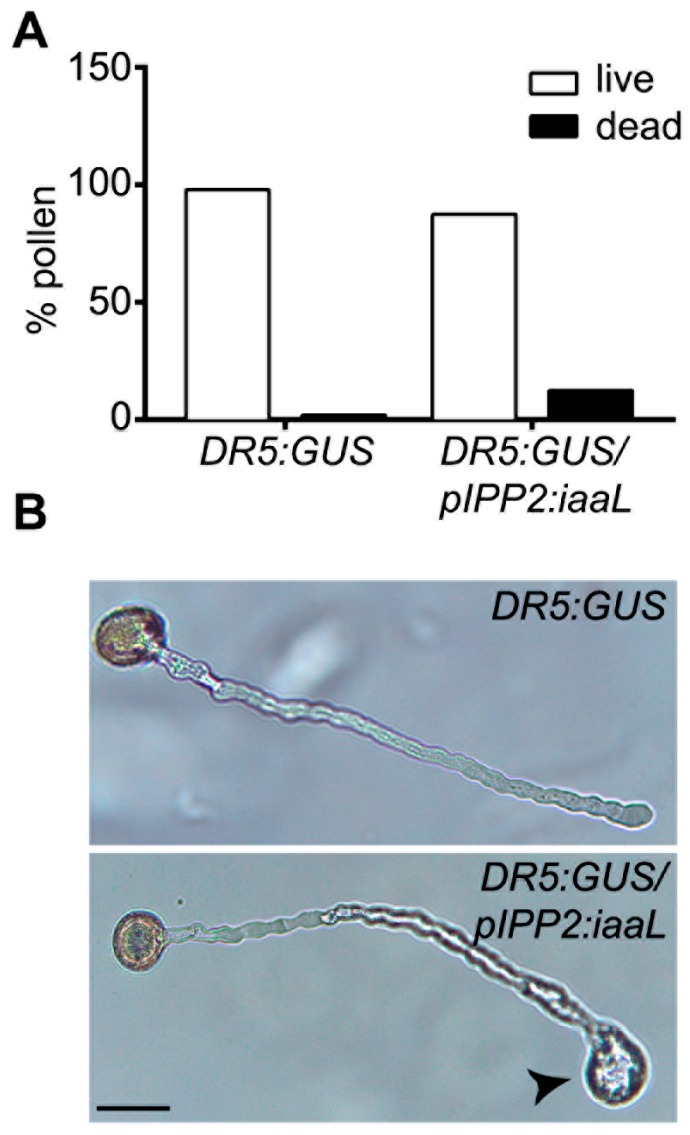
The pollen viability and the morphology of the pollen tube are affected in flowers of transgenic plants with a reduction in the auxin content. (**A**) Pollen viability analysis. Pollen from the DR5:GUS and DR5:GUS/_p_IPP2:*iaaL* transgenic plants was stained with Alexander staining, and was then observed and counted using microscopy techniques. The percentages of dead and live pollen are represented as black and white bars, respectively. (**B**) Representative images of pollen germination assays from DR5:GUS and DR5:GUS/_p_IPP2:*iaaL* plants. Pollen was germinated according to the conditions described in the methods section. The arrowhead indicates the aberrant balloon-shaped tip. Scalebar: 20 μm. The data were analyzed using a Fisher’s exact test comparing the genotypes. No significant differences were found (*p* = 0.0594, *n* = 50).

**Figure 6:**
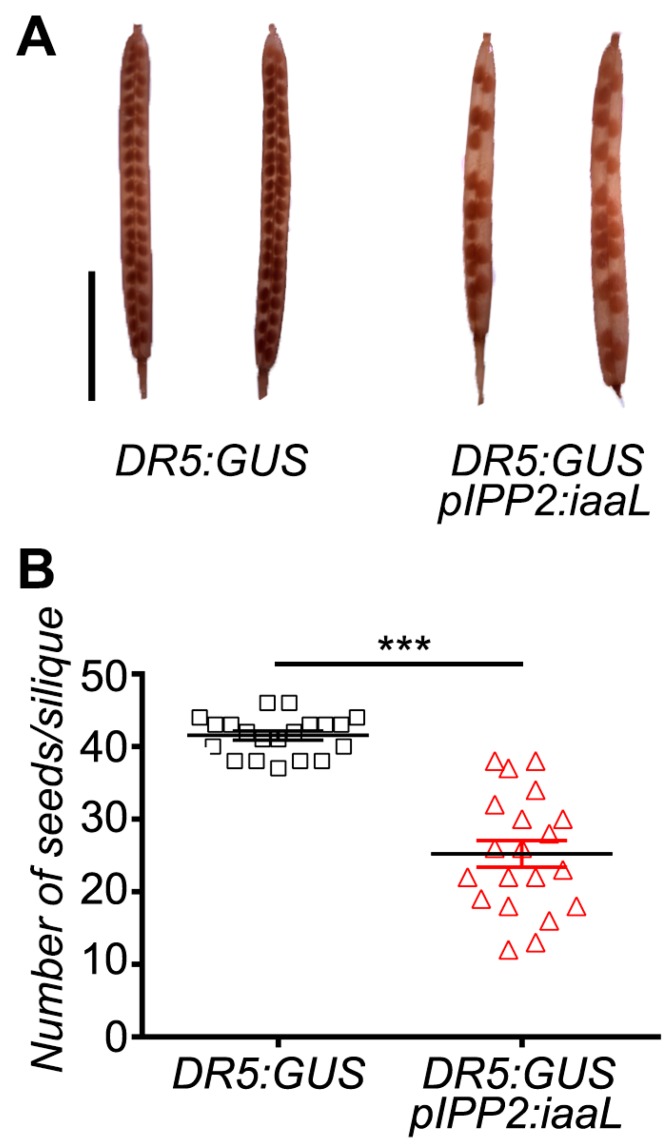
The reduction in auxin content in plants DR5:GUS/_p_IPP2:*iaaL* affects the number of seeds per silique. Quantification of seed content was performed in DR5:GUS/_p_IPP2:*iaaL* plants and the control DR5:GUS plants. Pictures of representative siliques are shown (**A**, scalebar: 5μm) and the number of seeds per silique was quantified. (**B**) Analysis was performed in 20 siliques of each genotype. Asterisks represent statistical differences between the genotypes according to an unpaired *t*-test (*p* < 0.001).

Next, a phenotypic analysis focusing on floral organs was carried out in flowers of transgenic _p_IPP2:*iaaL* plants, showing a reduced number of stamina (Figure 4). In all the evaluated flowers without a stamen, one of the short stamina was always missing. In addition, these plants also had a higher percentage of non-viable, dead pollen (Figure 5A), as well as defects in the elongation of the pollen tube tip compared to pollen from DR5:GUS plants (Figure 5B). Moreover, the siliques of _p_IPP2:*iaaL* transgenic plants contained a significantly reduced number of seeds when compared with the control plants (Figure 6). Altogether, these results suggest that the pollen-specific reduction of auxin affects both the development of stamina and viable pollen.

### 2.3. The Synchronization of Anther Dehiscence Is Altered in _p_IPP2:iaaL Plants

**Figure 7:**
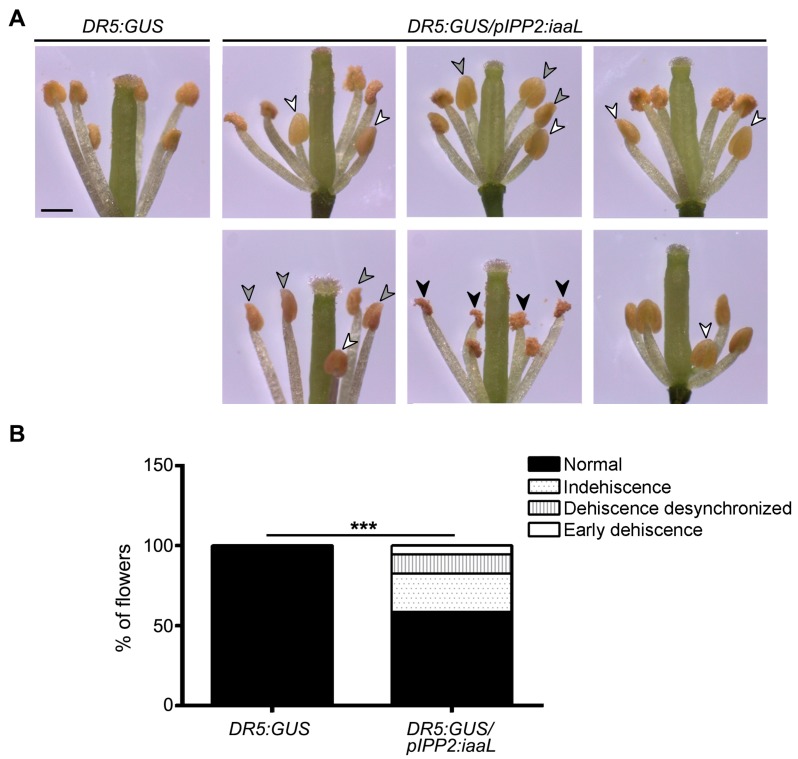
Reduction in flower auxin content via the expression of _p_IPP2*:iaaL* gene construct produces altered phenotypes in the dehiscence synchronization. (**A**) Pictures of DR5:GUS/_p_IPP2:*iaaL* flowers with different problems of dehiscence synchronization. A DR5:GUS flower is shown as a control. The arrowheads indicate different dehiscence problems as early dehiscence (black arrowheads), indehiscent anthers (gray arrowheads), and desynchronized dehiscence (white arrowheads). (**B**) Different defects in dehiscence synchronization were observed and quantified in DR5:GUS/_p_IPP2:*iaaL* and control DR5:GUS plants. The data are expressed as the percentage of normal flowers (black box), flowers with indehiscence (dotted white box), flowers with desynchronized dehiscence (dashed white box), and flowers with early dehiscence (white box). A total of 200 flowers per genotype were analyzed. Asterisks indicate significant differences between genotypes according to a Fisher’s exact test (*p* < 0.001).

It was reported that auxin is also an important determinant in the regulation of anther dehiscence [4]. Flowers of transgenic _p_IPP2:*iaaL* plants displayed a perturbation in the timing of anther dehiscence (Figure 7). The anthers of the flowers of _p_IPP2:*iaaL* transgenic plants showed earlier dehiscence, indehiscent anthers, dehiscence of only one anther, and dehiscence in immature anthers, all of which were not observed in control DR5:GUS plants. The observed perturbations of anther dehiscence in _p_IPP2:*iaaL* suggest the existence of retrograde signaling via which pollen contributes to the synchronization of anther dehiscence, likely through the control of auxin levels in pollen grains.

## 3. Discussion

The role of auxin and its importance during floral development were previously extensively described [4,6,10]. Nevertheless, to date, there are no studies connecting the accumulation of auxin in pollen grain and its potential role during development. The accumulation of auxin in pollen grain was previously described [6], but whether or not this accumulation occurs during different stages of pollen development was never investigated. Using histological analysis of flower buds during different stages, we indirectly observed that there is a differential accumulation of auxin in pollen over the course of development, which is independent of the contribution of anther tissues that surround the pollen. The accumulation of auxin can be observed throughout the entire development of the pollen, from early to late stages (Figure 1). We further investigated if this auxin accumulation in the male gametophyte plays a decisive role during flower development.

To decipher the role of auxin accumulation during pollen development, a genetic approach was chosen in which the levels of IAA were manipulated in a tissue-specific manner using the bacterial iaaL enzyme, which catalyzes the conjugation of IAA to l-lysine, thereby generating an inactive form of IAA [8]. Four promoters with different expression timing during pollen development were used to manipulate the expression of the *iaaL* gene in the pollen of *Arabidopsis thaliana* (Figure S2). The selected promoters were designated as early pollen promoter (from the sugar transporter 2 gene, *STP2*, At1g07340), intermediate pollen promoter 1 (from the sugar transporter 9 gene, *STP9*, At1g50310), intermediate pollen promoter 2 (from the pollen-specific gene 2 gene, *PSG2*, At1g28550), and late pollen promoter (from the phosphatase and tensin homolog deleted on chromosome 10 gene, *PTEN1*, At5g39400) [18,19,20,21]. The resulting constructs were transformed into the DR5:GUS genetic background, to facilitate the analysis of IAA accumulation by monitoring the expression of the reporter gene *GUS* under the control of the synthetic auxin-responsive DR5 promoter element [12]. Using this strategy, we were able to reduce free IAA levels in the transgenic _p_IPP2:*iaaL* plants (Figure 2). The feasibility of this strategy was previously proven [11], expressing the *iaaL* gene under the control of the promoter of the phosphatidylinositol monophosphate 5-kinase 1 (*PIP5K1*) gene. This construct directs the expression of *iaaL* gene to the stamina filament, decreasing the auxin content in stamina and consequently affecting the normal growth and development of these floral organs [11]. The pollen grain-specific expression of the pollen-specific gene 2 (*PSG2*) gene was previously reported as a pollen-specific promoter with exclusive activity in this floral organ [20]. The described transcriptional properties of *PSG2* promised that the utilization of this promoter was suitable to trigger an increased conjugation rate of IAA in pollen grain through the induced activity of the gene product of the linked *iaaL* gene, which, in turn, can be expected to reduce the amount of bio-active free IAA. Intriguingly, it was possible to detect a significant decrease in free IAA in flowers during an intermediate floral developmental stage (Figure 3, stage II: 9–10) [17], which is consistent with the spatiotemporal expression of the _p_*PSG2* (IPP2) promoter and also with the peak concentration of auxin in floral organs during development, as described previously [22]. In contrast, no differences in free IAA were detected at early and late developmental stages (represented by stages I and III) [17].

The reduced auxin levels in the transgenic _p_IPP2:*iaaL* plants, resulted in developmental defects in organs related to reproduction, such as the stamina and anthers (Figure 4), pollen grains (Figure 5), and seeds (Figure 6). The _p_IPP2:*iaaL* plants exhibited problems in the development of short stamina. The proper anatomy of stamina (four long and two short stamina) is required to successfully complete the fertilization process in *Arabidopsis* [15,16]. It was reported that a lack of an auxin transporter, such as *PIN6*, produces defective short stamina or even the loss of these structures, a phenotype also observed in _p_IPP2:*iaaL* transgenic lines, suggesting that the reduction in auxin accumulated in pollen affects the development of the stamina [23].

It is known that auxin is a pivotal plant hormone for proper control of plant development, including flower development and especially floral tissue maturation [6]. IAA contributes to the regulation of the development and elongation of stamina, and the maturation of the anthers and pollen [6]. For this reason, it can be concluded that the alteration of auxin levels in flowers is likely to affect dehiscence. The developments of anthers and pollen represent highly synchronized processes [6,15,16]. Hence, by decreasing the amount of auxin in the pollen grain, it may be possible to affect the maturation and dehiscence of the anther, perturbing the synchronization of the linked processes and generating defects in anther breaking and pollen release. Transgenic _p_IPP2:*iaaL* plants, which show a decrease in the auxin levels, display perturbations in the synchronization of anther dehiscence (Figure 7). The flowers of this transgenic line showed 41.5% of synchronization defects, including non-mature anthers, premature anthers, or anthers filled with water, which eventually do not lignify or break to release the pollen grain [1,5,24], suggesting that an auxin reduction, particularly in pollen, affects the correct anther dehiscence.

The observed alterated phenotypes, such as a reduction in the number of stamina, seed content, and defective dehiscense in two independent _p_IPP2:*iaaL* transgenic lines, positively correlated with the reduction in auxin signaling as monitored by GUS activity assays (Figure S5).

Furthermore, it is known that auxin is a driver of cell expansion, including pollen tube elongation [4,25]. Diminishing the amount of auxin in the pollen is supposed to affect the normal cylindrical shape of the pollen tube, resulting in aberrant balloon-shaped tips [26,27]. The _p_IPP2:*iaaL* pollen presented this phenotype in the tip of the pollen tube, suggesting that the reduction in auxin can affect the correct pollen tube formation (Figure 5). Furthermore, _p_IPP2:*iaaL* plants also presented a lower number of seeds compared to their control, DR5:GUS (Figure 6). This could be explained by the reduction in auxin present in the pollen, which ultimately affects the reproductive fitness of the plant similarly to the observation made in mutant plants lacking the *PIN8* gene, a pollen-specific auxin efflux carrier [8,9]. Therefore, it appears reasonable to conclude that the reduction in free auxin levels, specifically in pollen, can significantly affect the development of both the pollen grain and the elongation of the pollen tube, thereby affecting the reproduction and the fecundation rate of ovules, having an impact on the quantity of seeds in these transgenic plants.

The present work provides the first evidence for a central role of auxin accumulation in the pollen grain and its retrograde impact on stamina development via still unknown mechanisms which regulate the correct development of anthers and affect pollen germination and seed production.

## 4. Materials and Methods

### 4.1. Plant Growing

*Arabidopsis thaliana* ecotype Columbia (Col-0), DR5:GUS, and pPOLLEN:*iaaL* seeds were surface-sterilized for 6 min with a solution of 50% (*v*/*v*) commercial bleach, and then washed with sterile distilled water. Seeds were germinated and grown on solid Murashige and Skoog (MS) medium (Phytotechnology Laboratories) [28] containing 1% sucrose (*w*/*v*) and 0.8 (*w*/*v*) Phytagel (Sigma-Aldrich, St. Louis, MO, USA). After two weeks, plants were transferred to soil (Top Crop^TM^ Complete Mix, Santiago, Chile) and cultivated under a 16-h light/8-h dark cycle, at 23 °C. The pPOLLEN:*iaaL* plant seeds were grown and selected in a medium supplemented with BASTA (glufosinate ammonium) at a final concentration of 30 μg/mL.

### 4.2. β-Glucuronidase (GUS) Assay and Histological Analysis

The GUS assay was performed according to Reference [29]. Briefly, inflorescences were submerged in the GUS base solution buffer (100 mM NaH_2_PO_4_, pH 7.2; 10 mM EDTA, pH 8.0; 10% (*v*/*v*) methanol; 0.3% Triton X-100; 0.5 mM K_3_Fe(CN)_6_; 0.5 mM K_4_Fe(CN)_6_) supplemented with 1 mM 5-bromo-4-chloro-3-indolyl β-d-glucuronide (X-Gluc). A vacuum of 40 mPa was applied for 15 min. Thereafter, the plant tissues were incubated for 12 h at 37 °C to facilitate the enzymatic conversion of the substrate 5-bromo-4-chloro-3-indolyl β-d-glucuronide (X-gluc) to 5-bromo-4-chloro-3-indolyl, which was then oxidized and dimerized to give rise to the final insoluble reaction product (indigo). After the incubation, the tissues were washed with 70% ethanol until cleared. For the histological analysis, the GUS assay was performed and fixed in 2.5% glutaraldehyde in 0.1 M sodium cacodylate buffer (pH 7) overnight. After these procedures, the samples were sent to the Service of Microscopy of the Universidad Catolica de Chile for the histological sections. The sections were analyzed using an optical microscope Leica model ICC50 HD (Leica, Wetzlar, Germany) and images were captured employing the computer program Leica Acquire (Leica, Wetzlar, Germany), with a magnification of 100× with immersion oil.

### 4.3. Statistical Analysis of Data

The data from the statistical analysis are represented as means with standard error (SE) (Figure 3) and SD (Figure 6). Statistical comparisons between groups was performed using a Student’s *t*-test (Figure 6), Fisher’s exact test (Figure 4, Figure 5 and Figure 7), and two-way ANOVA/Bonferroni post test (Figure 3) employing the Prism 6 software package (GraphPad Software, San Diego, California, CA, USA). Differences were considered statistically significant for *p* < 0.05.

### 4.4. Cloning of Promoters and the iaaL Gene

The plasmid containing the *iaaL* gene was kindly provided by Dr. Lars Østergaard of the John Innes Center, Norwich. The promoters were cloned from genomic DNA extracted from *Arabidopsis thaliana* ecotype Columbia (Col-0). The DNA was extracted using the CTAB protocol [30]. The primers to amplify the promoters were as follows (F—forward, R—reverse): _P_AtSTP2 F: 5′-CACCAAACTCATTGCTTTCTCCTGA-3′; _P_AtSTP2 R: 5′-TGTTGTTGATCTCTTAGCTTCT-3′; _P_AtSTP9 F: 5′-CACCTGAGATTTAATGTGATGGT-3′; _P_AtSTP9 R: 5′-TTATTTATTCTTCACTTATTGAT-3′; _P_PSG2 F: 5′-CACCGGAAGAATACGAAGAAATAGTTGGC-3′; _p_PSG2 R: 5′-CTTATTTCCGAAATAAACCTTTTTGC-3′; _P_AtPTEN1 F: 5′-CACCGTGATCTGAGAAATGAGAGATTAC-3′; _P_AtPTEN1 R: 5′-TCTGAAGGAAGAAAACATATCATTA-3′; iaaL F: 5′-CACCATGACTGCCTACGATAATGGA-3′; and iaaL R: 5′-TCAGTTTCGGCGGTCGAT-3′.

The PCR products were cloned into pENTR^TM^(Thermo-Fisher, Waltham, Massachusetts, MA, USA) according to the manufacturer’s instructions. For the multiple recombination of fragments included in entry vectors, the enzyme mixture LR clonase^TM^ II plus and the destination vector pB7m24GW,3 were used according to the manufacturer’s protocol.

### 4.5. Reverse Transcriptase-Polymerase Chain Reaction (RT-PCR)

To perform the RT-PCR on RNA extracted from mature pollen grains, we used the RNeasy Plant Mini Kit (Qiagen, Hilden, Germany) and cDNA was obtained using the SuperScript II First-Strand Synthesis System (Invitrogen). Pollen cDNA was used as a template for the RT-PCR. The amplification of target genes was achieved using a Phusion High-Fidelity DNA polymerase (Thermo-Fisher, Waltham, Massachusetts, MA, USA), according to the manufacturer’s instructions, performing 24 cycles. PCR products were then separated on Lafken brand 1% agarose gels. The primers used for RT-PCR were as follows: RT-GUS F: 5’-ATGTTACGTCCTGTAGAAACCCCAACC-3’; RT-GUS R: 5’-TGTTCGGCGTGGTGTAGAGCATTA-3’; RT-iaaL F 5’-CACCATGACTGCCTACGATAATGGA-3’; RT-iaaL R 5’-TCAGTTTCGGCGGTCGAT-3’; EF1α-1 F: 5’-TCACCCTTGGTGTCAAGCAGAT-3’; and EF1α-1 R: 5’-CAGGGTTGTATCCGACCTTCTT-3’.

### 4.6. Floral Organ Analysis

The optical inspection of stamina, anthers, and pollen was performed using a stereo microscope Leica model EZ4HD (Leica, Wetzlar, Germany). Images were captured through the software Leica Acquire (Leica, Wetzlar, Germany).

### 4.7. Alexander Stain

The Alexander stain was performed according to Reference [31]. After the staining, the pollen was analyzed employing an optical microscope Leica model ICC50HD (Leica, Wetzlar, Germany) and photographs were taken using the software Leica Acquire (Leica, Wetzlar, Germany), with a magnification of 100× with immersion oil.

### 4.8. Pollen In Vitro Analysis

For in vitro germination tests of *A. thaliana* pollen grains, a solid germination medium containing 20% sucrose (*w*/*v*), 5 mM KCl, 5 mM CaCl_2_, 1 mM MgSO_4_, and 0.01% H_3_BO_3_ (*w*/*v*) was used. The pH of the medium was adjusted to 7.5 using a KOH solution, before being filtered. Then, low-melting agarose (Invitrogen) was added to a final concentration of 1.5% (*w*/*v*). After an incubation of 16 h in a humidity chamber, pollen tube growth was analyzed using optical microscopy Leica model ICC50HD (Leica, Wetzlar, Germany). Images were taken using the Leica Acquire software (Leica, Wetzlar, Germany).

### 4.9. Seed Analysis

Siliques of transgenic lines were collected and fixed in 100% ethanol. After the tissue lost its coloration, the siliques were inspected using optical microscopy Leica model EZ4HD (Leica, Wetzlar, Germany) and photos were taken using the Leica Acquire software (Leica, Wetzlar, Germany). Subsequently, the number of seeds in the inspected siliques was counted.

### 4.10. Auxin Analysis

Flowers from DR5:GUS and IPP2:*iaaL* plants were classified according to four different floral developmental stages (I: 8–9, II: 10–11 and III: 12–13) [17]. For each sample, flowers (40 mg) were harvested and shock-frozen in liquid nitrogen and stored at −80 °C. Auxin extraction was carried out as previously described [32]. In brief, 1 mL of pre-warmed (65 °C) methanol was added to each sample. The extraction proceeded for another 60 min at room temperature under gentle shaking. Each sample was spiked with 50 pmol of (^2^H_2_)-IAA as a stable isotope-labeled internal standard. After centrifugation (1 min, 12,000 rpm), supernatants were transferred into fresh micro-reaction tubes and dried under vacuum. In order to pre-purify the samples for subsequent gas chromatography–tandem mass spectrometry analysis (GC–MS/MS), the dry extracts were dissolved in 50 µL of methanol and 200 µL of diethyl ether. Thereafter, they were loaded onto aminopropyl solid-phase extraction cartridges. Each cartridge was washed twice with 250 µL of CHCl_3_:2-propanol (2:1, *v*/*v*). Next, the IAA-containing fraction was eluted with 400 µL of acidified diethyl ether (2% acetic acid, *v*/*v*). Thereafter, the eluates were transferred into 0.8-µL autosampler vials, and dried again in a gentle stream of nitrogen. Prior to mass spectrometric analysis, samples were derivatized by adding 20 µL of a mix consisting of 220 µL of acetone:methanol (9:1, *v*/*v*), 27 µL of diethyl ether, and 3 µL of a (trimethylsilyl)diazomethane solution (2.0 M in diethyl ether). To ensure complete derivatization, samples were incubated for 30 min at room temperature. Settings for the gas chromatograph and the mass spectrometer were as described previously [32]. For IAA analysis, the following transitions were recorded: MeIAA, *m*/*z* 189 to *m*/*z* 130 (quantifier ion), and *m*/*z* 130 to *m*/*z* 103 (qualifier ion); (^2^H_2_)-MeIAA, *m*/*z* 191 to *m*/*z* 132 (quantifier ion), and *m*/*z* 132 to *m*/*z* 103 (qualifier ion). The measurements were done in triplicate and at least three samples for each floral stages was analyzed, before the amount of endogenous hormone content was calculated from the signal ratio of the unlabeled to the stable isotope-containing mass fragment observed in the parallel measurements.

## Supplementary Materials

Supplementary materials can be found at http://www.mdpi.com/1422-0067/19/9/2480/s1.

## Abbreviations

IAA	3-indole acetic acid
*iaaL*	Indole acetic acid–lysine synthetase
_p_EPP	Early pollen promoter
_p_IPP	Intermediate pollen promoter
_p_IPP2	Intermediate pollen promoter 2
_p_LPP	Late pollen promoter
NPA	1-N-naphthylphthalamic acid
