# Supplementary material for: Modulation of Auxin Levels in Pollen Grains Affects Stamen Development and Anther Dehiscence in Arabidopsis
Source: Int J Mol Sci. 2018 Aug 22;19(9):2480. doi: 10.3390/ijms19092480 (PMC6164920; doi:10.3390/ijms19092480)
Supplement: Supplementary file 1 [file ijms-19-02480-s001.pdf]

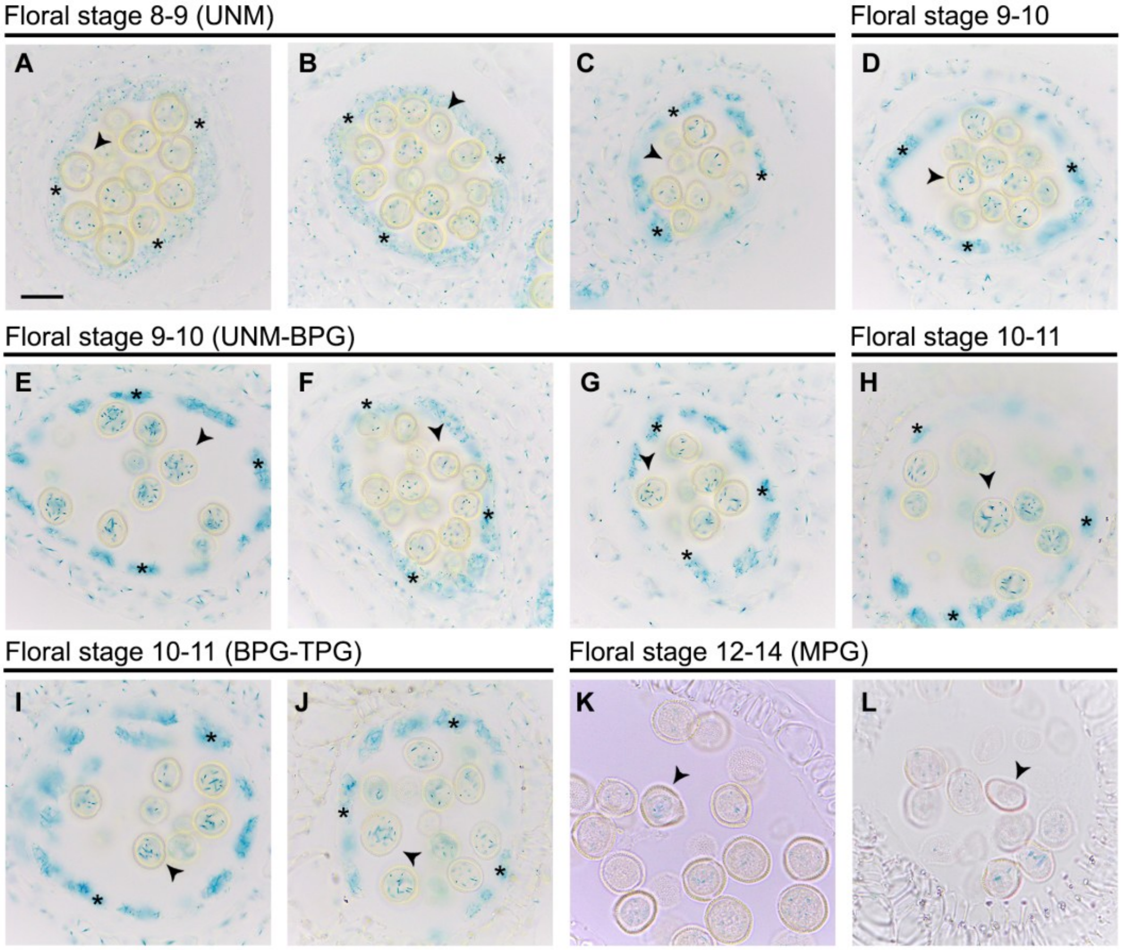

Supplementary Figure 1: The auxin transcriptional reporter DR5:GUS is expressed in different stages of pollen development. Histological sections of flowers from DR5:GUS plants in different stages of pollen grain development were incubated with the GUS substrate. **A**–**L** correspond to different stages of maturity of both anther and pollen grain from less mature (**A**) to a late and mature stage (**L**). Floral developmental stages and pollen developmental stages are indicated [14,17]. UNM: Uninucled Microspore; BPG: Bicellular pollen grain; TPG: tricellular pollen grain; MPG: Mature pollen grain. Tapetum and some pollen grains are indicated with asterisks and arrowheads, respectively. Scalebar: 20 μm.

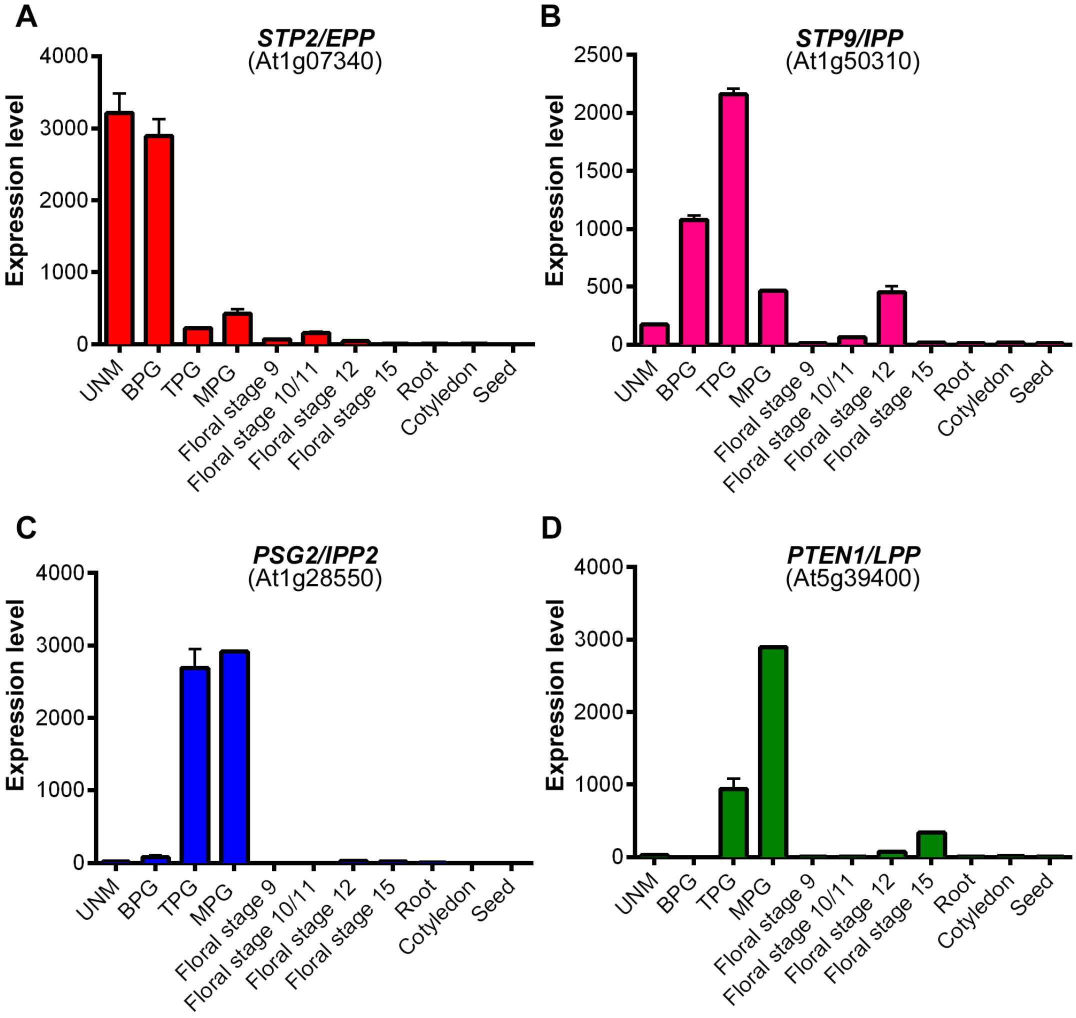

Supplementary Figure 2. Expression of genes STP2/EPP (At1g07340, **A**), STP9/IPP (At1g50310, **B**), PSG2/IPP2 (At1g28550, **C**) and PTEN1/LLP (At5g39400, **D**) in different tissues and stages of pollen development. Data obtained from EFp-Browser (Winter D.; Vinegar B.; Nahal H.; Ammar R.; Wilson G.V. and Provart N.J. An "Electronic Fluorescent Pictograph" Browser for Exploring and Analyzing Large-Scale Biological Data Sets. *PLoS ONE*. 2007, Volume: 2(8), e718. UNM: Uninucled Microspore; BPG: Bicellular pollen grain; TPG: tricellular pollen grain; MPG: Mature pollen grain.

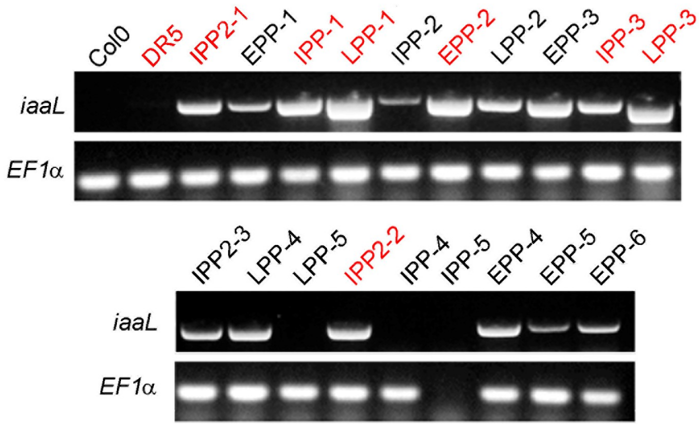

Supplementary Figure 3: The *iaaL* gene controlled by the different pollen specific promoters is expressed in pollen. To evaluate gene expression RNA from pollen was extracted and cDNA was synthetized. PCR was performed using specific oligonucleotides to detect the iaaL gene and EF1a as housekeeping gene. Every electrophoresis line represents different transgenic lines (EPP: early pollen

promoter, IPP: intermediate pollen promoter, LPP: late pollen promoter). The red highlighted genotypes indicate the transgenic lines presented in the manuscript.

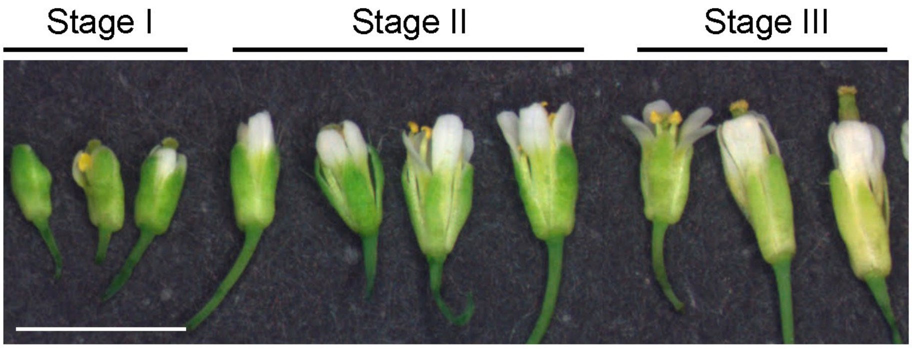

Supplementary Figure 4: Floral buds were classified in different stages for auxin measurement. According to the classification of Bowman et al. 1994 [17], the stage I include the phase 7–8, the stage II: phase 9–10 and stage III: phase 11–13. Scalebar: 2 mm.

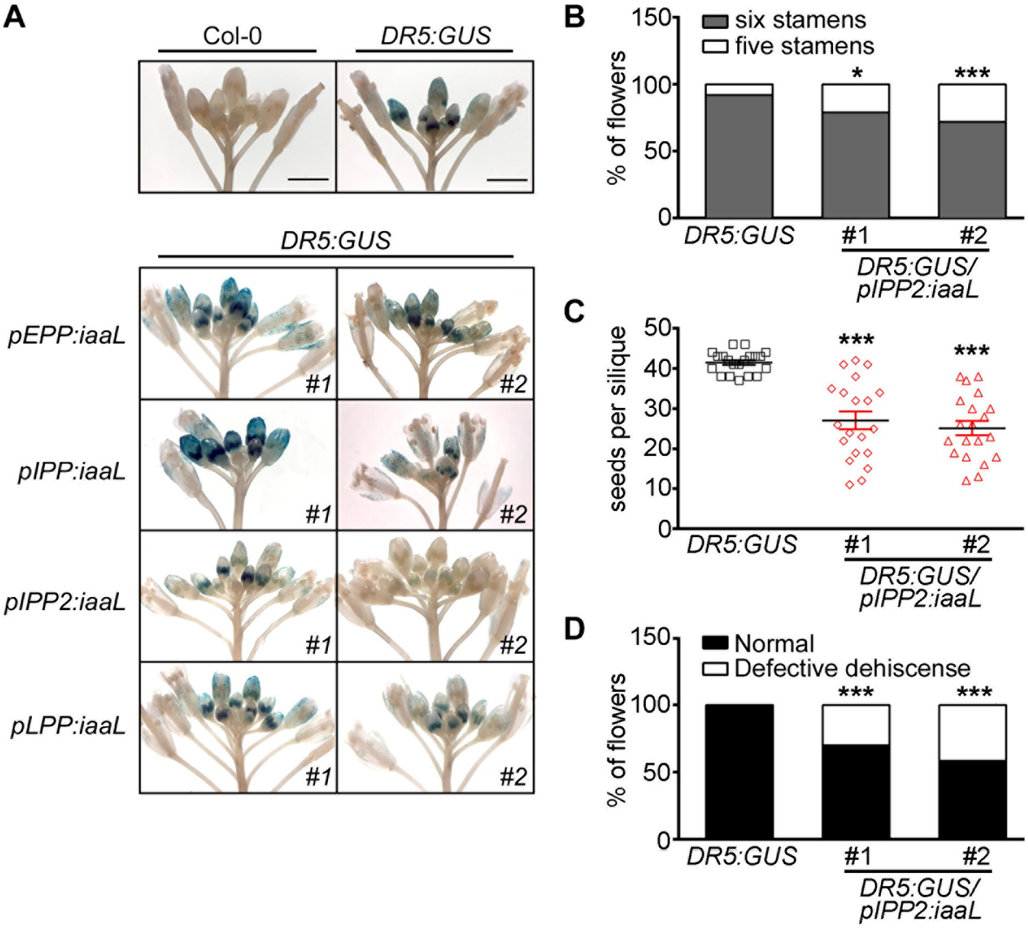

Supplementary Figure 5: Two independent DR5:GUS/pIPP2:iaaL lines with decrease in the auxin signaling display aberrant phenotypes. **A**. For each construction, two independent lines (indicated as #1 and #2) were obtained and the GUS stained inflorescences are shown. The line #2 is shown in the figures 2–7 in main text manuscript. (Scalebar: 20 mm). **B**–**D**: Floral phenotypic analysis of DR5:GUS/pIPP2:iaaL lines was performed: Number of stamina per flower (**B**), seeds per silique (**C**) and defective dehiscence (**D**) was analyzed quantified in 100 flowers per genotype (**B and D**) and 20 siliques (**C**). Asterisks indicate statistically significant differences compared to the control DR5:GUS transgenic line according to a Fischer's exact test (**B**,**D**) and unpaired *t-test* (**C**) (* $p < 0.05$, *** $p < 0.001$).
